# A certificateless aggregate signature scheme for VANETs with privacy protection properties

**DOI:** 10.1371/journal.pone.0317047

**Published:** 2025-02-20

**Authors:** Huimin Li, Chucheng Shen, Hui Huang, Chenhuang Wu

**Affiliations:** 1 Fujian Key Laboratory of Financial Information Processing, Putian University, Fujian, Putian, China; 2 Key Laboratory of Financial Mathematics of Fujian Province University, Putian University, Fujian, Putian, China; 3 Fujian ZhongXin Network Security Information Technology Company Limited, Fujian, Fuzhou, China; 4 School of Computer Science, Minnan Normal University, Fujian, Zhangzhou, China; National University of Sciences and Technology, UNITED KINGDOM OF GREAT BRITAIN AND NORTHERN IRELAND

## Abstract

Aggregate signatures are excellent in simultaneously verifying the validity of multiple signatures, which renders them highly suitable for bandwidth-constrained environments. The certificateless public key system is among the most advanced public key cryptosystems at present. Scholars have combined their advantages to develop certificateless aggregate signature schemes, which are applicable to the secure communication of Vehicular Ad-hoc Networks (VANETs). Recently, Cahyadi E F et al. put forward a certificateless aggregate signature scheme specifically designed for use in VANETs. Regrettably, through our strict security analysis, we discovered at least two major vulnerabilities in the signature scheme: a public key replacement attack and a malicious KGC (Key Generation Center) attack. To tackle these vulnerabilities, our article not only presents the methods of these attacks but also explores the fundamental reasons for their feasibility. Additionally, we propose specific improvement measures and show that the enhanced scheme retains its security under the random oracle model. The stability of the improved scheme depends on the computational complexity of the Diffie-Hellman problem. Finally, a comprehensive assessment involving security, computational cost, communicational cost, and calculational efficiency overhead highlights the excellent performance of our proposed solution.

## 1 Introduction

The global increase in car ownership has led to a higher risk of traffic safety issues, such as accidents and congestion. Vehicular Ad-hoc Networks (VANETs) utilize advanced information technology to establish an extensive network connection among entities, facilitating real-time monitoring of vehicle flow and driving conditions [1]. This innovative approach aims to mitigate or even prevent traffic accidents, enhance traffic efficiency, and optimize the overall driving environment. The core components of VANETs typically consist of three entities: a Trusted Authority (TA), vehicles equipped with On-Board Units (OBUs), and Roadside Units (RSUs). VANETs enable vehicles to broadcast messages every 100 to 300 milliseconds via the Dedicated Short-Range Communication (DSRC) protocol [2]. These frequent broadcasts enable vehicles to make well-informed decisions based on the received information. For example, they can choose faster and safer travel routes based on real-time transmission data to relieve traffic congestion, minimize or avoid traffic accidents, and enhance road utilization, traffic efficiency. Moreover, VANETs provide a variety of value-added services like navigation, media, and social media, thereby remarkably enhancing people’s driving experience [3, 4].

However, the open and broadcast nature of VANETs’ communication facilitates real-time information exchange but also makes it vulnerable to various security threats like message forgery and tampering [5]. In VANETs, vehicle users depend on public communication channels which are usually insecure. Consequently, this vulnerability enables adversaries to intercept and manipulate information, possibly resulting in the exposure of vehicle users’ identity details. These security breaches might also allow the tracking of vehicle users’ travel patterns, thus endangering the safety of individuals and their property. Furthermore, there is the risk of malicious vehicles deliberately spreading false information to disrupt traffic flow for their gain. For instance, a vehicle may broadcast false reports of congestion ahead, causing other vehicles to avoid specific routes during route planning and giving the malicious vehicle an unfair edge. In such cases, it is essential for trustworthy entities to quickly verify the identities of the malicious entities in the network. Therefore, strong conditional privacy protection and message authentication mechanisms are required in VANETs. Moreover, considering the limited communication bandwidth in VANETs, these authentication processes need to be designed to reduce communication overhead as much as possible.

The certificateless public key cryptosystem [6], which was first introduced by Al Riyami et al. at ASIACRYPT 2003, is a high-performance and versatile cryptographic framework and has become one of the most advanced public key systems available since then. It doesn’t need certificates and still keeps robust security. In the field of digital signatures, the concept of aggregate signatures, which was initially presented by Boneh et al. [7] at EUROCRYPT 2003, has proved to be particularly innovative. This approach enables multiple signatures to be consolidated into a single (aggregated signature), thus simplifying the verification process. The verifier only needs to confirm the validity of this aggregated signature to verify the authenticity of all individual signatures. Therefore, aggregate signatures have broad application value in network communication environments that have low computational load, low bandwidth, low capacity, and require mutual verification of signature validity. It is essential to explore a certificateless aggregate signature scheme which combines the advantages of both certificateless cryptography and aggregate signatures, because such a scheme provides the dual benefits of conditional anonymity and enhanced security. And it is well-suited for applications that require efficient network communication and mutual verification of signature integrity, like healthcare wireless medical sensor networks (HWMSNs) and VANETs. In recent years, scholars have been highly interested in enhancing the security and privacy of networks, including devising certificateless aggregate signature schemes for HWMSNs and VANETs. For example, Zhou et al. [8] proposed an improved pair-free certificateless aggregate signature scheme for HWMSNs which was based on the work of Zhan et al. [9]. Altaf et al. [10] proposed a privacy-preserving local hybrid authentication scheme that is suitable for large vehicle networks. Almazroi et al. [11] proposed an efficient certificateless authentication scheme for fog computing in 5G-assisted vehicle systems. Xu et al. [12] proposed a certificateless aggregate signature scheme aimed at ensuring secure routing. Wang et al. [13] utilized full aggregation technology to reduce computing and bandwidth resources, proposed a certificateless aggregate signature scheme for implementing conditional privacy protection in VANETs and proved the security of the scheme under the standard model. Liang et al. [14] analyzed and improved an efficient certificateless aggregate signature scheme that has conditional privacy protection properties and is applied to VANETs. Yu et al. [15] proposed the first scheme for aggregation on a vehicle-mounted unit. This scheme performed a certificateless aggregate signature on the vehicle-mounted unit to avoid channel conflicts. Moreover, they utilized elliptic curve point multiplication operations to reduce computational costs. Rajkumar et al. [16] put forward an improved scheme that ensures robust privacy protection and security. They utilized aggregation and elliptic curve point addition to enhance efficiency without affecting verification speed or adding extra costs for roadside units.

Although several certificateless aggregate signature schemes suitable for VANETs already exist, some of them have not been subject to rigorous security analysis and might be vulnerable to attacks like public key replacement and KGC(Key Generation Center) attacks. For example, Jiu et al. [17] pointed out that the scheme proposed by Thumbur et al. [18] is prone to signature forgery attacks and then proposed a new certificateless aggregate signature scheme without pairing based on [18]. Similarly, Zhou et al. [19] pointed out that neither the scheme of Thumbur et al. [18] nor that of Chen et al. [20] can resist public key replacement attacks, and proposed an improved certificateless aggregate signature scheme which is more suitable for resource-constrained VANETs environments. Shim et al. [21] also indicated that the scheme of Chen Y [20] is vulnerable to forgery attacks and Type I attacks. Ali et al. [22] presented a certificateless aggregate signature scheme with provable security, providing conditional privacy authentication within a random oracle model framework. However, Zhou et al. [23] emphasized that the scheme of Ali et al. is vulnerable to malicious vehicle attacks or malicious KGC attacks. Xiong et al. [24] proposed a certificateless aggregate signature scheme based on elliptic curve cryptography which can resist collusion attacks. However, Xu et al. [25] pointed out that this scheme can be forged by Type I adversaries. Wu et al. [26] pointed out that the pair-free certificateless aggregate signature scheme with conditional privacy protection proposed by Gong et al. [27] for VANETs fails to achieve the required unlinkability and is vulnerable to public key replacement forgery attacks by Type I adversaries. In 2022, Cahyadi et al. [28] developed a certificateless aggregate signature scheme with security and privacy protection for VANETs. Unfortunately, through analysis, we found that this signature scheme is also insecure. This paper will analyze and indicate that the signature scheme is vulnerable to at least two types of attacks, and offer a detailed analysis of the specific reasons for the existence of these attack methods. Additionally, corresponding improvement measures to defend against these attacks are proposed, and the enhanced security of the improved signature scheme is demonstrated.

The layout of this article is organized as follows. The related work is discussed in Section 2. Essential preliminary knowledge is presented in Section 3. A comprehensive review of the certificateless aggregate signature scheme proposed by Cahyadi et al. is shown in Section 4. Subsequently, Section 5 explores the vulnerabilities of Cahyadi et al.’s scheme, illustrating two distinct attack methodologies. In Section 6, we first analyze the underlying causes of these attacks. Then, we introduce an improved certificateless aggregate signature scheme which is designed to address the aforementioned vulnerabilities. This new scheme is accompanied by rigorous security proof, demonstrating its robustness. Section 7 focuses on an analysis of computational and communication costs, assessing the efficiency of our proposed scheme. Finally, Section 8 presents the conclusion and future work.

## 2 Related work

In the open wireless communication environment of VANETs, several crucial security challenges emerge, mainly concerning the dynamic authentication of vehicles, the efficiency and effectiveness of authentication procedures, and the location privacy issues of vehicles. Ensuring the security and protecting the privacy of users have become crucial issues in the research field of VANETs [29, 30].

To ensure the integrity and reliability of messages received in VANETs and prevent malicious actors from tampering with communications or impersonating legitimate users, researchers have adopted reputation management systems. These systems allow vehicles to evaluate the trustworthiness of their peers and the received messages, thus reducing the risks related to deceptive messages spread by malicious entities [31–33]. To assess the reliability of sensor vehicles, some schemes like those in literature [34–36] have carried out real-time updates of reputation values. Reputation update is a crucial part of reputation management. It is typically performed regularly by Trusted Authority (TA) after gathering, decrypting, and verifying a large amount of reputation feedback. This leads to high computational and communication costs for the TA and may even cause the TA to become a bottleneck in the reputation management system [33, 37]. Moreover, digital signature technology is also a significant technology for guaranteeing the security of VANETs. It ensures that communication messages between entities are signed for security. In the communication process, the sender encrypts the information using a private key, and the receiver verifies the signature first to confirm the reliability of the message source. Furthermore, to improve the efficiency of verification processes and reduce communication overhead, VANETs security protocols frequently utilize aggregate signature technology and certificateless signature technology. These methods are preferred due to their advantages in optimizing signature efficiency and decreasing the overall system overhead. Then, certificateless aggregate signature schemes have been continuously proposed for VANETs [12–28] (A brief introduction to these algorithms can be found in Section 1.).

On the other hand, privacy protection is also a crucial aspect of research in VANETs. Without privacy protection for reputation feedback, sensitive information about vehicle privacy may be leaked. When a dispute over messages occurs, it is expected that only trusted institutions can track and extract the true identity of the message sender, thus achieving conditional privacy protection for user identity. To address such issues, Liu et al. [34] proposed a scheme that can simultaneously offer accurate trust management and robust conditional privacy protection by using the well-known Private Set Intersection (PSI) technique based on Bloom Filter (BF). Cheng et al. [31] designed a lightweight privacy-preserving sensing task-matching algorithm. This algorithm is implemented by devising algorithms to verify the validity of reputation values, selecting reliable sensing vehicles, and an efficient reputation management mechanism that can update the reputation values of sensing vehicles efficiently and accurately. So, this algorithm can protect location privacy, identity privacy, sensing data privacy, and reputation value privacy while decreasing the computational and communication overhead of sensing vehicles. Liu et al. [38] proposed a cloud-assisted vehicle network privacy protection reputation update (PPRU) scheme based on elliptic curve cryptography (ECC) and the Parlier algorithm. In this scheme, reputation feedback is provided by honest but curious cloud service providers (CSPs). Moreover, the pseudonym mechanism is also a means of protecting user privacy. Zhou et al. [39] proposed a certificateless aggregate signature scheme, which uses full aggregation technology to reduce computation and bandwidth resources and implements conditional privacy protection through a pseudonymization mechanism to ensure the communication security and privacy protection of VANETs. References [13–15, 20, 22, 26, 27] also combine the pseudonym mechanism with the certificateless aggregate signature to ensure the conditional privacy of VANETs. Furthermore, the utilization of algorithms incorporating privacy principles represents a means of safeguarding user privacy. Shahrouz et al. [40] proposed an anonymous authentication scheme based on zero-knowledge proof to achieve conditional privacy protection of VANETs. Mundhe et al. [41] proposed a conditional privacy protection authentication scheme based on lightweight ring signatures and pseudonyms for VANETs. Zhang [42] proposed a privacy protection announcement scheme based on message link group signatures for VANETs. This scheme has information-binding and efficient revocation functions and exhibits strong credibility.

In the domain of communication security and privacy preservation in VANETs, a promising research approach is to integrate reputation management, pseudonymization mechanisms, and advanced digital signatures technologies like certificateless aggregate signatures, ring signatures, and group signatures. This approach not only improves network security but also reduces communication and storage costs. Moreover, it incorporates a fault-tolerant mechanism that ensures operational continuity even when a trusted authority is absent or there is a partial infrastructure disruption, thereby offering a practical solution for building a reliable and trustworthy vehicle network.

## 3 Preliminary

### 3.1 Bilinear mapping and its properties

Let *G*_1_ and *G*_2_ denote two groups respectively, where *G*_1_ is an additive group with an order of a large prime number *q*, *G*_2_ is a multiplication group having the same order *q*. *P* is a generator of *G*_1_. Then the bilinear pairing operation is defined as a bilinear mapping $\hat{e}:G_{1}\times G_{1}\to G_{2}$ that satisfies the following properties:

1. Bilinear: Let *P*,*Q*,*R*∈*G*_1_, the equation $\hat{e}(P+R,Q)=\hat{e}(Q,P+R)=\hat{e}(Q,P)\hat{e}(Q,R)$ and $\hat{e}(aP,bQ)=\hat{e}(bQ,aP)=\hat{e}(abP,Q)=\hat{e}(P,abQ)=\hat{e}{\left(P,Q\right)}^{ab}$ hold for any $a,b\in Z_{q}^{*}$.
2. Nondegeneracy: $\hat{e}(P,P)\neq 1_{G_{2}}$.
3. Computability: For any *P*,*Q*∈*G*_1_, an efficient algorithm can be found to compute $\hat{e}(P,Q)$.

It is known that $a,b\in Z_{q}^{*}$, *P*,*Q*,*aP*,*bP*∈*G*_1_, there are two Difficult Problems in Group *G*_1_:

1. Discrete Logarithm Problem (DLP): Finding an integer *n* that satisfies *Q* = *nP* is difficult.
2. Computational Diffie-Hellman Problem (CDHP): Calculating *abP*∈*G*_1_ is difficult.

### 3.2 Two types of attacks in certificateless cryptosystems

In the certificateless cryptosystem, the authentication of a personal public key does not rely on the certificate issued by the certificate authority (CA). The user private key comprises two parts: the secret value selected by the user and the partial private key generated by KGC for the user. Therefore, in a certificateless public key system, it is commonly assumed that an attacker will replace the user’s public key with a randomly chosen value to assess the security of the system. That is, it is assumed that the attacker already knows the user’s secret value at this stage. Furthermore, as the user’s partial private key is generated by KGC, it is assumed that KGC is untrustworthy. This implies that if the attacker knows the system’s master private key, they can generate the user’s partial private key. It is assumed that, of course, an attacker cannot possess the aforementioned capabilities simultaneously. Otherwise, the attacker would have the user’s private key and could naturally perform all actions that the user can. For this reason, there are two types of attackers in the security model of certificateless public key systems [1], namely type I (denoted as *A*_I_) and type II (denoted as *A*_II_). Although *A*_I_ does not know the system master private key, it can replace the user’s public key and then it knows the user’s secret value. *A*_II_ cannot replace the user’s public key, but it can calculate the user’s partial private key because it knows the system’s master private key. In practical applications, *A*_I_ simulates attackers other than KGC, while *A*_II_ simulates malicious KGC attackers.

A secure signature scheme in a certificateless system must be capable of withstanding the previously mentioned attacks. Moreover, regardless of whether it is under the attack of the first type attacker *A*_I_ or the second type attacker *A*_II_, the signature scheme must be unforgeable.

In order to characterize the attacking capabilities of attacker *A*_I_ and attacker *A*_II_, it is usually carried out in the following Game I and Game II respectively. In view of Game I and Game II, if the success probability of an attacker *A* (including *A*_I_ and *A*_II_) in forging a signature can be negligible, then this certificateless aggregate signature scheme is secure.

*A*_I_ and *A*_II_ can access the following six oracles:

1. *CreateUser*: This oracle returns the vehicle’s public key $vpk_{{\mathrm{PID}}_{i}}$ to *A* after receiving the vehicle’s pseudo-identity PID_*i*_.
2. *RevealPartialPrivateKey*: This oracle returns the vehicle’s partial private key $Psk_{{\mathrm{ID}}_{i}}$ to *A* after receiving the vehicle’s real identity ID_*i*_.
3. *RevealPrivateKey*: This oracle returns the vehicle’s private key $vsk_{{\mathrm{PID}}_{i}}$ to *A* after receiving PID_*i*_.
4. *RevealPseudonym*: The challenger *C* searches the list *L*_*K*_ when *A* requests the pseudonym of the vehicle ID_*i*_. If the entry exists, this oracle uses PID_*i*_ to respond. Otherwise, it returns ⊥.
5. *ReplaceKey*: This oracle will update the vehicle’s $vpk_{{\mathrm{PID}}_{i}}$ to $vpk_{{\mathrm{PID}}_{i}}^{\prime }$ after receiving the new public key $vpk_{{\mathrm{PID}}_{i}}^{\prime }$ selected by *A* and PID_*i*_.
6. *Sign*: The oracle machine returns a signature *σ*_*i*_ regarding the message *M*_*i*_ to *A* after receiving PID_*i*_ and *M*_*i*_∈{(0,1)*}.

Game I:

Setup: Challenger *C* inputs the security parameter *l* to generate master key *s* and public parameters *params*. Then *C* keeps *s* confidential, and sends *params* to *A*_I_.

Query: *A*_I_ is allowed to run several oracles such as *CreateUser*, *RevealPrivateKey*, *RevealPartialPrivateKey*, *ReplaceKey* and *Sign*.

Forgery: A signature *σ*′ on message *M*′ is generated by *A*_I_ finally. Where *M*′ is the message of the target identity PID* whose public key is $vpk_{\mathrm{ID}*}$.

If *A*_I_ achieves the following points when challenging identity PID*, it is considered that *A*_I_ has won

**Game I**.

1. *A*_I_ did not conduct partial private key $Psk_{{\mathrm{ID}}^{*}}$ inquiries regarding the target user’s identity PID*
2. *A*_I_ did not inquire about the signature signed by the target user’s identity PID* on *M*′.
3. *σ*′ is about the valid signature of $vpk_{\mathrm{ID}*}$ and PID* on *M*′.

**Game II**:

Setup: Challenger *C* inputs the security parameter *l* to generates master key *s* and public parameters *params*. Then *C* sends *s* and *params* to *A*_II_.

Query: *A*_II_ is allowed to run several oracles such as *CreateUser*, *RevealPrivateKey* and *Sign*. Because *A*_II_ can access *s*, it no longer needs *RevealPartialPrivateKey*.

Forgery: A signature *σ*′ on the message *M*′ is generated by *A*_II_ finally, where *M*′ is the message of the target identity PID* whose public key is $vpk_{\mathrm{ID}*}$.

If *A*_II_ achieves the following points, it is considered that *A*_II_ has won the Game II.

1. *A*_II_ did not conduct private key $vsk_{\mathrm{ID}*}$ inquiries regarding the target user’s identity PID*.
2. *A*_II_ did not inquire about the signature signed by target user’s identity PID* on the message *M*′.
3. *σ*′ is about the valid signature of $vpk_{\mathrm{ID}*}$ and PID* on *M*′.

## 4 Restatement of Cahyadi et al.’s certificateless aggregate signature scheme

Cahyadi et al.’s certificateless aggregate signature scheme consists of nine algorithms as follows [28]:

**Setup:** KGC inputs safety parameter *l*∈*N*, selects large prime numbers *q*, constructs an additive group *G*_1_ and a multiplication group *G*_2_, where *G*_1_ and *G*_2_ have the same order *q*. Then KGC selects a generator *P* from *G*_1_, and selects a bilinear pairwise mapping $\hat{e}:G_{1}\times G_{1}\to G_{2}$. The system master and the private key are selected randomly as $\alpha \in Z_{q}^{*}$, and the system public key is calculated as *P*_*pub*_ = *αP*. KGC selects six secure one-way hash functions $h_{1},h_{2},\cdots h_{6}:{\left\{0,1\right\}}^{*}\to Z_{q}^{*}$.

Meanwhile, TRA (Trace Authority) selects a secret value $\beta \in Z_{q}^{*}$, then calculates public key *T*_*pub*_ = *βP*. Every RSU in the vehicle self-organizing network needs to randomly select a number $y_{i}\in Z_{q}^{*}$ as their private key, then calculates the corresponding public key $P_{rsu_{i}}=y_{i}P$, and sends $P_{rsu_{i}}$ to KGC. Common system parameters $params=\{G_{1},G_{2},q,\hat{e},P_{pub},h_{1},h_{2},\cdots ,h_{6},P_{rsu_{i}}\}$ will be sent to each vehicle in the network by KGC after KGC received $P_{rsu_{i}}$, and *params* will also be preloaded on TPD(Tamper-Proof Device).

**Registration:** Each vehicle equipped with OBU devices needs to register with the TRA. Thus, the TRA is capable of tracking the false identities of vehicles and precisely identifying which vehicle used a false identity in disputable situations. The registration process comprises three steps:

Firstly, TRA selects a hash function $H(\cdot ):{\left\{0,1\right\}}^{*}\to G_{1}$.

Then, TRA selects a random number $r_{i}\in Z_{q}^{*}$ and the vehicle’s identity ID_*i*_, associating it with the new actual identity $Q_{{\mathrm{ID}}_{i}}=H({\mathrm{ID}}_{i},r_{i})$ which will be used in all further communication in the future. $\left\{Q_{{\mathrm{ID}}_{i}},r_{i}\right\}$ will be transmitted to the vehicle through a secure communication channel by TRA. After receiving this information, the vehicle selects *nonce* which is a random number, and subsequently computes the password $pass_{{\mathrm{ID}}_{i}}=h_{1}(r_{i},nonce)$.

Finally, $pass_{{\mathrm{ID}}_{i}}$ is sent to TRA via a secure communication channel, TRA stores $pass_{{\mathrm{ID}}_{i}}$, and vehicle registration is completed.

**Partial-Private-Key-Gen:** KGC uses the vehicle’s identity $Q_{{\mathrm{ID}}_{i}}$ and system master private key *α* to generate its partial private key $Psk_{{\mathrm{ID}}_{i}}=\alpha Q_{{\mathrm{ID}}_{i}}$. Secure communication channels are used by KGC to send $Psk_{{\mathrm{ID}}_{i}}$ to the vehicle. The vehicle can verify $Q_{{\mathrm{ID}}_{i}}$ and $Psk_{{\mathrm{ID}}_{i}}$ by Eq (1) to determine whether they are created by legal TRA and KGC.


$$\hat{e}(Psk_{{\mathrm{ID}}_{i}},P)=\hat{e}(Q_{{\mathrm{ID}}_{i}},P_{pub}).$$ (1)

**Vehicle-Key-Gen:** To obtain a pseudo-identity from the TRA, a vehicle must first follow the steps below to generate its own public and private keys.

①The vehicle randomly selects a value $x_{i}\in Z_{q}^{*}$ as its private key $vsk_{{\mathrm{ID}}_{i}}$, that is $vsk_{{\mathrm{ID}}_{i}}=x_{i}$.
②The vehicle calculates its public key $vpk_{{\mathrm{ID}}_{i}}=x_{i}P$ by using the common parameter *P*.
③The vehicle calculates a one-time password $OTP=h_{2}(pass_{{\mathrm{ID}}_{i}}⊕Q_{{\mathrm{ID}}_{i}}⊕T_{i})$ used for mutual authentication with TRA based on the current timestamp *T*_*i*_, $pass_{{\mathrm{ID}}_{i}}$ and $Q_{{\mathrm{ID}}_{i}}$, and this *OTP* can only be used once. Afterward, the vehicle encrypts the message $<E_{T_{pub}}(m,OTP,{\mathrm{ID}}_{i},T_{i},vpk_{{\mathrm{ID}}_{i}})>$ by using the public key cryptography system and *T*_*pub*_, and sends the encrypted message to TRA, where *m* represents that the vehicle’s OBU device stores *m* pseudo identities.
④Using its private key *β*, the TRA decrypts the received message $<E_{T_{pub}}(m,OTP,{\mathrm{ID}}_{i},T_{i},vpk_{{\mathrm{ID}}_{i}})>$ and checks if *T*_*i*_ falls in a valid time period. If so, TRA calculates $Q_{{\mathrm{ID}}_{i}}^{\circ }=H({\mathrm{ID}}_{i},r_{i})$ and $OTP^{\circ }=h_{2}(pass_{{\mathrm{ID}}_{i}}⊕Q_{{\mathrm{ID}}_{i}}^{\circ }⊕T_{i})$. If *OTP*^∘^ = *OTP*, TRA can verify the vehicle’s identity.

**Pseudonym-Gen:** TRA will produce a pseudo-identity PID_*i*_ for the vehicle upon successful mutual authentication between the TRA and the vehicle.

①TRA creates a pseudo-identity ${\mathrm{PID}}_{i}=C_{i}Q_{{\mathrm{ID}}_{i}}$ by calculating $C_{i}=h_{3}(\beta ,{\mathrm{ID}}_{i},T_{i}^{\circ })$.
②TRA calculates *D*_*i*_ = *h*_4_(PID_*i*_) and *K*_*i*_ = *C*_*i*_*D*_*i*_.

The vehicle has to preload several pseudo-identities and ensure that none of them are reused. Whenever a message is sent to another vehicle, a different pseudo-identity is used. When vehicles are in areas where many vehicles congregate, like intersections or parking lots, this approach will make it hard for legitimate vehicles to be tracked by malicious attackers. The TRA decides the number of pseudo-identities to generate based on the current number of pseudo-identities stored in the OBU device. Each application process leads to the generation of multiple pseudo-identities $\left({\mathrm{PID}}_{i,1},{\mathrm{PID}}_{i,2},\cdots ,{\mathrm{PID}}_{i,n}\right)$.

③TRA calculates $j=h_{5}(Q_{{\mathrm{ID}}_{i}},T_{i}^{\circ })$, and encrypts messages *M*_1_ (Eq 2) by using $vpk_{{\mathrm{ID}}_{i}}$.

$$M_{1}=\langle E_{vpk_{{\mathrm{ID}}_{i}}}(({\mathrm{PID}}_{i,1},K_{i,1}),({\mathrm{PID}}_{i,2},K_{i,2}),\cdots ,({\mathrm{PID}}_{i,n},K_{i,n}),j,T_{i}^{\circ })\rangle .$$ (2)

RSU sends *M*_1_ to the vehicle. Then the vehicle will use $vsk_{{\mathrm{ID}}_{i}}$ to decrypt the message (Eq 3) after receiving the *M*_1_, and verify if $T_{i}^{\circ }$ is in a valid time interval. If so, the vehicle calculates $j^{\circ }=h_{5}(Q_{{\mathrm{ID}}_{i}},T_{i}^{\circ })$ and verifies if *j*^∘^ = *j* holds.


$$\langle D_{vsk_{{\mathrm{ID}}_{i}}}(E_{vpk_{{\mathrm{ID}}_{i}}}(({\mathrm{PID}}_{i,1},K_{i,1}),({\mathrm{PID}}_{i,2},K_{i,2}),\cdots ,({\mathrm{PID}}_{i,n},K_{i,n}),j,T_{i}^{\circ }))\rangle .$$ (3)

After successful verification, the vehicle and the TRA will conduct mutual authentication. Subsequently, the vehicle will store the pseudo-identities together with *K*_*i*_. Since OBU devices must store sufficient pseudo-identities, vehicles need to reapply to the TRA at appropriate times to prevent a lack of pseudo-identities.

**Sign:** ①The vehicle selects a random number $u_{i}\in Z_{q}^{*}$, and calculates *U*_*i*_ = *u*_*i*_*P*∈*G*_1_.

②OBU calculates *h*_*i*_ (Eq 4) and *S*_*i*_ (Eq 5) to form a certificateless signature *σ*_*i*_ = (*U*_*i*_,*S*_*i*_) for the message *M*_*i*_.

$$h_{i}=h_{6}(M_{i},{\mathrm{PID}}_{i},vpk_{{\mathrm{ID}}_{i}},U_{i},t_{i}).$$ (4)

$$S_{i}=K_{i}Psk_{{\mathrm{ID}}_{i}}+vsk_{{\mathrm{ID}}_{i}}P_{pub}+h_{i}u_{i}P_{rsu_{i}}.$$ (5)

③Vehicle broadcast final message $\left(M_{i},{\mathrm{PID}}_{i},vpk_{{\mathrm{ID}}_{i}},\sigma_{i},t_{i}\right)$, where *t*_*i*_ is the timestamp.

**Verify:** This step is executed by RSU. When the RSU detects a message sent by the vehicle, it first verifies *t*_*i*_. If the message is not signed within an acceptable time frame, *t*_*i*_ will not be accepted and the message will be abandoned. Conversely, RSU calculates $h_{i}=h_{6}(M_{i},{\mathrm{PID}}_{i},vpk_{{\mathrm{ID}}_{i}},U_{i},t_{i})$ and *D*_*i*_ = *h*_4_(PID_*i*_). Finally, RSU verifies Eq (6). If the verification is successful, the certificateless signature is accepted. Conversely, if the verification fails, it implies potential message tampering and the signature will not be accepted.


$$\hat{e}(S_{i},P)=\hat{e}(D_{i}PID_{i}+vpk_{{\mathrm{ID}}_{i}},P_{pub})\cdot \hat{e}(h_{i}U_{i},P_{rsu_{i}}).$$ (6)

**Aggregate:** When RSU receives *n* message signature pairs $\left(M_{1},\cdots ,M_{n},\sigma =(U_{1},\cdots ,U_{n},S_{1},\cdots ,S_{n}),t_{1},\cdots ,t_{n}\right)$ from the vehicle with pseudo-identities (PID_1_,⋯,PID_*n*_). It calculates the formula $S=\sum_{i=1}^{n}S_{i}$ and returns *σ* = (*U*_1_,⋯,*U*_*n*_,*S*) as an aggregate signature.

Finally, RSU sends $\left(M_{1},\cdots ,M_{n},{\mathrm{PID}}_{1},\cdots ,{\mathrm{PID}}_{n},vpk_{{\mathrm{ID}}_{1}},\cdots ,vpk_{{\mathrm{ID}}_{n}},\sigma ,t_{1},\cdots ,t_{n}\right)$ to AS.

**Aggregate-verify:** When AS receives *σ* = (*U*_1_,⋯,*U*_*n*_,*S*) from RSU, it calculates $h_{i}=h_{6}(M_{i},{\mathrm{PID}}_{i},vpk_{{\mathrm{ID}}_{i}},U_{i},t_{i})$ and $D_{i}=h_{4}({\mathrm{PID}}_{i}),i=1,2,\cdots ,n$. Finally, the AS verifies if the Eq (7) holds. If so, the aggregate signature is valid and is accepted. Otherwise, it is invalid and is rejected.


$$\hat{e}(S,P)=\hat{e}(\sum_{i=1}^{n}(D_{i}\cdot {\mathrm{PID}}_{i}+vpk_{{\mathrm{ID}}_{i}}),P_{pub})\cdot \hat{e}(\sum_{i=1}^{n}h_{i}U_{i},P_{rsu_{i}}).$$ (7)

## 5 Two attack methods for Cahyadi et al.’s certificateless aggregate signature schemes

The subsequent analysis reveals that the certificateless aggregate signature scheme presented in [28] is vulnerable to at least two distinct malicious attacks, as detailed below:

1. Attack Method 1: KGC Malicious Attack
  KGC starts with the signature algorithm **Sign** to attack. Firstly, it is noticed that in the signature algorithm **Sign**, the calculated formula is equivalently deformed as follows:
$$S_{i}=Psk_{{\mathrm{ID}}_{i}}K+vsk_{{\mathrm{ID}}_{i}}P_{pub}+h_{i}u_{i}P_{rsu_{i}}$$
$$=\alpha Q_{{\mathrm{ID}}_{i}}C_{i}D_{i}+x_{i}\alpha P+h_{i}u_{i}P_{rsu_{i}}$$
$$=\alpha \cdot D_{i}\cdot {\mathrm{PID}}_{i}+\alpha \cdot vpk_{{\mathrm{ID}}_{i}}+h_{i}u_{i}P_{rsu_{i}}.$$
  The equation shows that KGC is capable of generating an authentic signature for any arbitrary message $M_{i}^{\prime }$ intended for any vehicle ID_*i*_, by using the system’s master private key *α*, even without access to the private key *x*_*i*_ of the specific vehicle. The specific attack method is described below:
  ① Choose any message $M_{i}^{\prime }$ to be signed.
  ② The vehicle selects a random number $u_{i}^{\prime }\in Z_{q}^{*}$, and calculates $U_{i}^{\prime }=u_{i}^{\prime }P\in G_{1}$.
  ③ OBU calculates $h_{i}^{\prime }$ (Eq 8) and $S_{i}^{\prime }$ (Eq 9) to form a certificateless signature $\sigma_{i}^{\prime }=(U_{i}^{\prime },S_{i}^{\prime })$ for the message $M_{i}^{\prime }$.
$$h_{i}^{\prime }=h_{6}(M_{i}^{\prime },{\mathrm{PID}}_{i},vpk_{{\mathrm{ID}}_{i}},U_{i}^{\prime },t_{i}^{\prime }).$$ (8)
$$S_{i}^{\prime }=\alpha (D_{i}\cdot {\mathrm{PID}}_{i}+vpk_{{\mathrm{ID}}_{i}})+h_{i}u_{i}^{\prime }P_{rsu_{i}}.$$ (9)
  The forgery method described above cannot be distinguished from a genuine signature generated by the legitimate signer with their private key in Cahyadi et al.’s signature protocol. Moreover, it meets the verification formula of the aggregate signature algorithm. Notably, when examining the verification formula for individual signatures, it can be seen that the forgery satisfies the verification Eq (6). That is because
$$\hat{e}(S_{i}^{\prime },P)=\hat{e}(\alpha (D_{i}\cdot {\mathrm{PID}}_{i}+vpk_{{\mathrm{ID}}_{i}})+h_{i}u_{i}^{\prime }P_{rsu_{i}},P)$$
$$=\hat{e}(\alpha (D_{i}\cdot {\mathrm{PID}}_{i}+vpk_{{\mathrm{ID}}_{i}}),P)\cdot \hat{e}(h_{i}u_{i}^{\prime }P_{rsu_{i}},P)$$
$$=\hat{e}(D_{i}\cdot {\mathrm{PID}}_{i}+vpk_{{\mathrm{ID}}_{i}},\alpha P)\cdot \hat{e}(h_{i}u_{i}^{\prime }P,P_{rsu_{i}})$$
$$=\hat{e}(D_{i}\cdot {\mathrm{PID}}_{i}+vpk_{{\mathrm{ID}}_{i}},P_{pub})\cdot \hat{e}(h_{i}U_{i}^{\prime },P_{rsu_{i}}).$$
  Therefore, KGC successfully forged a signature on any selected message $M_{i}^{\prime }$.
2. Attack Method 2: Public Key Replacement Attack
  The aggregate signature scheme proposed in reference [28] is based on a certificateless public key cryptography system. Thus, it is essential to consider replacement public key attacks. An attacker can successfully forge signatures on any selected message *M*′ by commencing with the verification algorithm, as described below:
  ① Chooses any message $M_{i}^{\prime }$ to be signed.
  ② Selects $l\in Z_{q}^{*}$, and replaces the vehicle public key with $vp{k_{{\mathrm{ID}}_{i}}}^{\prime }=lP-D_{i}\cdot {\mathrm{PID}}_{i}$.
  ③ The vehicle selects a random number $u_{i}^{\prime }\in Z_{q}^{*}$, let $U_{i}^{\prime }=u_{i}^{\prime }P\in G_{1}$.
  ④OBU calculates $h_{i}^{\prime }$ (Eq 10) and $S_{i}^{\prime }$ (Eq 11) to form a certificateless signature $\sigma_{i}^{\prime }=(U_{i}^{\prime },S_{i}^{\prime })$ for the message $M_{i}^{\prime }$.

$$h_{i}^{\prime }=h_{6}(M_{i}^{\prime },{\mathrm{PID}}_{i},vp{k_{{\mathrm{ID}}_{i}}}^{\prime },U_{i}^{\prime },t_{i}^{\prime }).$$ (10)

$$S_{i}^{\prime }=lP_{pub}+h_{i}u_{i}^{\prime }P_{rsu_{i}}.$$ (11)

⑤ Outputs the signature $\sigma_{i}^{\prime }=(U_{i}^{\prime },S_{i}^{\prime })$ of the message $M_{i}^{\prime }$.

The forgery method described above cannot be distinguished from a signature that would have been produced by the signer using the vehicle’s private key in Cahyadi et al.’s signature scheme. Moreover, it completely conforms to the verification Eq (6), which makes it hard to tell from an authentic signature. The reason is that

$$\hat{e}(S_{i}^{\prime }P)=\hat{e}(lP_{pub}+h_{i}u_{i}^{\prime }P_{rsu_{i}},P)=\hat{e}(lP_{pub},P)\cdot \hat{e}(h_{i}u_{i}^{\prime }P_{rsu_{i}},P)$$

$$=\hat{e}(lP,P_{pub})\cdot \hat{e}(h_{i}u_{i}^{\prime }P_{rsu_{i}},P)=\hat{e}(D_{i}\cdot {\mathrm{PID}}_{i}+vp{k_{{\mathrm{ID}}_{i}}}^{\prime },P_{pub})\cdot \hat{e}(h_{i}U_{i}^{\prime },P_{rsu_{i}}).$$

Therefore, the attacker successfully forged a signature on any selected message $M_{i}^{\prime }$.

## 6 Improvement methods and analysis to overcome the above attacks

### 6.1 Improvement methods to overcome the above attacks

Firstly, we will discuss how to overcome attack method 1(KGC Malicious Attack). Note that the reason why attack method 1 was successful is due to the computation *S*_*i*_, which involved $Psk_{{\mathrm{ID}}_{i}}K+vsk_{{\mathrm{ID}}_{i}}P_{pub}=\alpha Q_{{\mathrm{ID}}_{i}}C_{i}D_{i}+x_{i}\alpha P=\alpha \cdot D_{i}\cdot {\mathrm{PID}}_{i}+\alpha \cdot vpk_{{\mathrm{ID}}_{i}}$ in Eq (5). The way to overcome this attack is to change the generation method of *S*_*i*_, that is the vehicle’s secret value and private key need to be used separately. The fundamental reason why attack method 2 (Public Key Replacement Attack) can succeed is that PID_*i*_⋅*D*_*i*_ can be eliminated by a new public key $vp{k_{{\mathrm{ID}}_{i}}}^{\prime }=lP-D_{i}\cdot {\mathrm{PID}}_{i}$.

In addition, it was found that there was a conceptual error in the proof of their Theorem 1(See reference [28] for details) of Cahyadi et al.’s scheme, as follows:

$$(u_{i}^{\prime }bP^{\prime }+v_{i}^{\prime }aP^{\prime }+h_{i}^{\prime }cP^{\prime })-(u_{i}^{\prime }bP^{\prime }+v_{i}^{\prime }aP^{\prime }+h_{i}^{\prime *}cP^{\prime })=abc((u_{i}^{\prime }+v_{i}^{\prime }+h_{i}^{\prime })-(u_{i}^{\prime }+v_{i}^{\prime }+h_{i}^{\prime *}))P^{\prime }.$$

This is obviously not right. Therefore, we propose an improved signature scheme as follows:

**Setup:** KGC and TRA execute this algorithm to generate relevant parameters. The generation method of each parameter is the same as Cahyadi et al.’s signature scheme. KGC sends common system parameters $params=\{q,G_{1},G_{2},\hat{e},P_{pub},h_{1},h_{2},\cdots ,h_{6},P_{rsu_{i}}\}$ to each vehicle in the network, and secretly stores system master private key *α*.

**Registration:** TRA completes registration of vehicle equipped with OBU. This algorithm is equivalent to Cahyadi et al.’s signature scheme.

**Partial-Private-Key-Gen:** KGC executes this algorithm to create the vehicle’s partial private keys $Psk_{{\mathrm{ID}}_{i}}=\alpha Q_{{\mathrm{ID}}_{i}}$. This algorithm is the same as Cahyadi et al.’s signature scheme.

**Vehicle-Key-Gen:** The vehicle’s unique public and private keys will be created through this algorithm by the vehicle itself. This algorithm is identical to Cahyadi et al.’s signature scheme in terms of its functionality and output.

**Pseudonym-Gen:** TRA and the vehicle execute this algorithm to generate the vehicle’s pseudo-identity PID_*i*_. This algorithm is the same as Cahyadi et al.’s signature scheme.

**Sign:** While the fundamental principles of this algorithm remain similar to Cahyadi et al.’s signature scheme, a key difference lies in the modified method for generating *S*_*i*_. Vehicle ID_*i*_ generates a signature for message *M*_*i*_ in the following two steps:

①The vehicle selects a random number $u_{i}\in Z_{q}^{*}$, and calculates *U*_*i*_ = *u*_*i*_*P*∈*G*_1_.
②OBU calculates *h*_*i*_ (Eq 12) and *S*_*i*_ (Eq 13) to form a certificateless signature *σ*_*i*_ = (*U*_*i*_,*S*_*i*_) for the message *M*_*i*_, where *t*_*i*_ represents the timestamp.

$$h_{i}=h_{6}(M_{i},{\mathrm{PID}}_{i},vpk_{{\mathrm{ID}}_{i}},U_{i},t_{i}).$$ (12)

$$S_{i}=K_{i}Psk_{{\mathrm{ID}}_{i}}+(vsk_{{\mathrm{ID}}_{i}}+h_{i}u_{i})P_{rsu_{i}}.$$ (13)

③Vehicle broadcasts the final message $\left(M_{i},{\mathrm{PID}}_{i},vpk_{{\mathrm{ID}}_{i}},\sigma_{i},t_{i}\right)$.

**Verify:** This algorithm is executed by RSU, and it is basically the same as Cahyadi et al.’s signature scheme, but modify the validation formula to Eq (14).


$$\hat{e}(S_{i},P)=\hat{e}(D_{i}\cdot {\mathrm{PID}}_{i},P_{pub})\cdot \hat{e}(vpk_{{\mathrm{ID}}_{i}}+h_{i}U_{i},P_{rsu_{i}}).$$ (14)

**Aggregate:** This is an algorithm for generating an aggregate signature *σ* = (*U*_1_,⋯,*U*_*n*_,*S*) which is executed by RSU, and it is the same as Cahyadi et al.’s scheme.

**Aggregate-verify:** The AS utilizes the algorithm to authenticate the legitimacy of the signature. This algorithm is fundamentally identical to Cahyadi et al.’s signature scheme, necessitating only minor modifications to the verification formula to adapt it to the new context. AS verifies if the Eq (15) holds. If it holds, the aggregate signature is considered valid and accepted. Otherwise, it is invalid and rejected.


$$\hat{e}(S,P)=\hat{e}(\sum_{i=1}^{n}(D_{i}\cdot {\mathrm{PID}}_{i}),P_{pub})\cdot \hat{e}(\sum_{i=1}^{n}\left(vpk_{{\mathrm{ID}}_{i}}+h_{i}U_{i}\right),P_{rsu_{i}}).$$ (15)

### 6.2 Security analysis

#### 6.2.1 Correctness

Note that the verification of a single signature involves the following steps:

$$\hat{e}(S_{i},P)=\hat{e}(K_{i}Psk_{{\mathrm{ID}}_{i}}+(vsk_{{\mathrm{ID}}_{i}}+h_{i}u_{i})P_{rsu_{i}},P)$$

$$=\hat{e}(K_{i}Psk_{{\mathrm{ID}}_{i}},P)\cdot \hat{e}(x_{i}P_{rsu_{i}},P)\cdot \hat{e}(h_{i}u_{i}P_{rsu_{i}},P)$$

$$=\hat{e}(C_{i}D_{i}\alpha Q_{Id_{i}},P)\cdot \hat{e}(x_{i}P,P_{rsu_{i}})\cdot \hat{e}(h_{i}u_{i}P,P_{rsu_{i}})$$

$$=\hat{e}(D_{i}\cdot {\mathrm{PID}}_{i},P_{pub})\cdot \hat{e}(vpk_{{\mathrm{ID}}_{i}},P_{rsu_{i}})\cdot \hat{e}(h_{i}U_{i},P_{rsu_{i}})$$

$$=\hat{e}(D_{i}\cdot {\mathrm{PID}}_{i},P_{pub})\cdot \hat{e}(vpk_{{\mathrm{ID}}_{i}}+h_{i}U_{i},P_{rsu_{i}}).$$

The verification of the aggregate signature is as follows:

$$\hat{e}(S,P)=\hat{e}(\sum_{i=1}^{n}\left(K_{i}Psk_{{\mathrm{ID}}_{i}}+(vsk_{{\mathrm{ID}}_{i}}+h_{i}u_{i})P_{rsu_{i}}\right),P)$$

$$=\hat{e}(\sum_{i=1}^{n}K_{i}Psk_{{\mathrm{ID}}_{i}},P)\cdot \hat{e}(\sum_{i=1}^{n}x_{i}P_{rsu_{i}},P)\cdot \hat{e}(\sum_{i=1}^{n}h_{i}u_{i}P_{rsu_{i}},P)$$

$$=\hat{e}(\sum_{i=1}^{n}C_{i}D_{i}\alpha Q_{ID_{i}},P)\cdot \hat{e}(\sum_{i=1}^{n}x_{i}P,P_{rsu_{i}})\cdot \hat{e}(\sum_{i=1}^{n}h_{i}u_{i}P,P_{rsu_{i}})$$

$$=\hat{e}(\sum_{i=1}^{n}D_{i}\cdot {\mathrm{PID}}_{i},P_{pub})\cdot \hat{e}(\sum_{i=1}^{n}vpk_{{\mathrm{ID}}_{i}},P_{rsu_{i}})\cdot \hat{e}(\sum_{i=1}^{n}h_{i}U_{i},P_{rsu_{i}})$$

$$=\hat{e}(\sum_{i=1}^{n}D_{i}\cdot {\mathrm{PID}}_{i},P_{pub})\cdot \hat{e}(\sum_{i=1}^{n}vpk_{{\mathrm{ID}}_{i}}+h_{i}U_{i},P_{rsu_{i}}).$$

Therefore, the modified signature scheme is correct.

#### 6.2.2 Unforgeability

Theorems 1 and 2 shown below illustrate that, within the random oracle model, the enhanced signature scheme is resistant to attacks from adversaries *A*_I_ and *A*_II_, respectively.

**Theorem 1** Under the attack of adaptive choose identity and message in a random oracle model. If the attacker *A*_I_ can complete *q*_*H*_ times of *H* queries, $q_{h_{i}}$ times of *h*_*i*_ (*i* = 1,2,⋯,6) queries, *q*_*c*_ times of *q*_*c*_ queries, $q_{Psk_{{\mathrm{ID}}_{i}}}$ times of *RevealPartiaPrivatelKey* queries, $q_{vsk_{{\mathrm{ID}}_{i}}}$ times of *RevealPrivateKey* queries, *q*_*rp*_ times of *ReplaceKey* queries, *q*_*sign*_ times of *Sign* queries with A in time $t_{Succ_{A_{I}}}$, and successfully create a valid aggregate signature with a non-negligible probability. Then A can completely solve the CDH problem with an undeniable probability $\frac{\varepsilon }{q_{H}e^{2}}$ in time $t^{\prime }=t_{Succ_{A_{I}}}+(q_{c}+q_{H}+\sum_{i=1}^{6}q_{h_{i}}+q_{Psk_{{\mathrm{ID}}_{i}}}+q_{vsk_{{\mathrm{ID}}_{i}}}+q_{rp}+q_{sign})t_{M}$, where *t*_*M*_ denotes the duration necessary to perform scalar multiplication in the group *G*_1_ once, while *e* stands for a natural constant with a value of approximately 2.718281828459045.

**Proof** In the following section, we present a demonstration within Game I that illustrates the resilience of our CLAS scheme against forgery attacks launched by *A*_I_. Let A be the challenger to solve the CDH problem, and (*aP*,*bP*) be any instance of the CDH problem. The subsequent proof demonstrates that, assuming the random oracle model, if *A*_I_ is capable of successfully forgery a signature, the challenger A can exploit *A*_I_’s forgery capability to solve the CDH problem.

Firstly, A selects a challenge identity $I\hat{D}$ and maintains some lists to assist in answering the attacker *A*_I_’s inquiries in Game I. In this paper, we use * to indicate a wildcard character, and use ⊥ to indicate that the item is empty below.

Next, A and *A*_I_ will play the following game:

1. A runs algorithm *S*et*up*, defines system parameters $params=\{q,G_{1},G_{2},\hat{e},h_{i}(\cdot ),P_{pub},P_{rsu_{i}}\}$, and sends *params* to *A*_I_. Among them, let *P*_*pub*_ = *aP*, $P_{rsu_{i}}=kP$, $k\in Z_{q}^{*}$. We note that the output of the Hash function $H(\cdot ),h_{i}(\cdot ),i=1,2,\cdots 6$ is answered by A under the random oracle model. Then *A*_I_ begins to execute the following inquiry, and A answers *A*_I_’s related inquiries by maintaining the list $L_{H},L_{h_{1}},L_{h_{2}},L_{h_{3}},L_{h_{4}},L_{h_{5}},L_{h_{6}},L_{K}$ which are initialized as empty.
  *L*_*H*_ contains a triplet (ID_*i*_,*r*_*i*_,*l*_*i*_).
  $L_{h_{1}}$ contains a triplet $\left(r_{i},nonce,pass_{{\mathrm{ID}}_{i}}\right)$.
  $L_{h_{2}}$ contains a quadruple $\left(pass_{{\mathrm{ID}}_{i}},Q_{{\mathrm{ID}}_{i}},T_{i},OTP_{i}\right)$.
  $L_{h_{3}}$ contains a quadruple $\left(\beta ,{\mathrm{ID}}_{i},T_{i}^{\circ },C_{i}\right)$.
  $L_{h_{4}}$ contains a binary (PID_*i*_,*D*_*i*_).
  $L_{h_{5}}$ contains triplet $\left(Q_{{\mathrm{ID}}_{i}},T_{i}^{\circ },j_{i}\right)$.
  $L_{h_{6}}$ contains a six tuple $\left(M_{i},{\mathrm{PID}}_{i},vpk_{{\mathrm{ID}}_{i}},U_{i},t_{i},h_{i}\right)$.
  *L*_*K*_ contains a quintuple $\left({\mathrm{ID}}_{i},{\mathrm{PID}}_{i},Psk_{{\mathrm{ID}}_{i}},vsk_{{\mathrm{ID}}_{i}},vpk_{{\mathrm{ID}}_{i}}\right)$.
2. *CreateUser* query: Assuming *A*_I_ registers identity ID_*i*_ with A. A first check *L*_*H*_, if there is no record of (ID_*i*_,*,*) in *L*_*H*_, A makes *H* query with himself firstly. If *L*_*H*_ contains a record (ID_*i*_,*,*), then continue to check if *L*_*K*_ contains (ID_*i*_,*,*,*,*). If so, further confirm whether $vpk_{{\mathrm{ID}}_{i}}$ is ⊥. If so, A selects a random value $v_{i}\in Z_{q}^{*}$ as $vsk_{{\mathrm{ID}}_{i}}$, and computes $vpk_{{\mathrm{ID}}_{i}}=v_{i}P$. Then A sends $vpk_{{\mathrm{ID}}_{i}}$ to *A*_I_, and updates the corresponding element $\left(vpk_{{\mathrm{ID}}_{i}},vsk_{{\mathrm{ID}}_{i}}\right)$ in list *L*_*K*_. If $vpk_{{\mathrm{ID}}_{i}}\neq ⊥$, A directly sends $vpk_{{\mathrm{ID}}_{i}}$ to *A*_I_. Additionally, if *L*_*K*_ does not include (ID_*i*_,*,*,*,*), A performs *h*_3_ and *h*_4_ queries to establish a pseudo-identity and store it in the $L_{h_{4}}$ and *L*_*H*_ lists. Then A selects a random value $v_{i}\in Z_{q}^{*}$ as $vsk_{{\mathrm{ID}}_{i}}$, computes $vpk_{{\mathrm{ID}}_{i}}=v_{i}P$, sends $vpk_{{\mathrm{ID}}_{i}}$ to *A*_I_, and adds $\left({\mathrm{ID}}_{i},{\mathrm{PID}}_{i},Psk_{{\mathrm{ID}}_{i}},vsk_{ID_{i}},vpk_{ID_{i}}\right)$ to the list *L*_*K*_.
3. *RevealPartialPrivateKey* query: When *A*_I_ asks the partial private key of ${\mathrm{ID}}_{i},i\in [1,q_{psk}]$, A checks if ID_*i*_ is a challenge identity $I\hat{D}$. If so, A outputs failure and stops the simulation. Otherwise, A queries *L*_*H*_ to confirm whether (ID_*i*_,*r*_*i*_,*l*_*i*_) is already in the list *L*_*H*_, if so, returns *l*_*i*_*aP*.
4. *RevealPseudonym* query: When *A*_I_ asks the corresponding PID_*i*_ for ID_*i*_, A finds (ID_*i*_,*,*,*,*) in *L*_*K*_. If *L*_*K*_ contains (ID_*i*_,*,*,*,*), A confirms whether PID_*i*_ is ⊥. If PID_*i*_≠⊥, A returns PID_*i*_ to *A*_I_. Otherwise, A finds the corresponding record by querying *L*_*H*_. If not, self-inquire about the identity ID_*i*_ and record it. If ${\mathrm{ID}}_{i}=I\hat{D}$, let PID_*i*_ = *C*_*i*_*bP*, where *C*_*i*_ corresponds to the list $L_{h_{3}}=(\beta ,{\mathrm{ID}}_{i},T_{i}^{\circ },C_{i})$. Otherwise, let PID_*i*_ = *C*_*i*_*l*_*i*_*P*, where *l*_*i*_ is from the record (ID_*i*_,*r*_*i*_,*l*_*i*_) of *L*_*H*_. A returns PID_*i*_ to *A*_I_, and adds $\left({\mathrm{ID}}_{i},{\mathrm{PID}}_{i},Psk_{{\mathrm{ID}}_{i}},vsk_{ID_{i}},vpk_{ID_{i}}\right)$ to the list *L*_*K*_.
5. *RevealPrivateKey* query: When *A*_I_ asks the private key of ${\mathrm{PID}}_{i},i\in [1,q_{vsk}]$, A checks whether $\left({\mathrm{ID}}_{i},{\mathrm{PID}}_{i},Psk_{{\mathrm{ID}}_{i}},vsk_{ID_{i}},vpk_{ID_{i}}\right)$ is included in *L*_*K*_. If $vpk_{{\mathrm{ID}}_{i}}=⊥$, A makes a *CreateUser* query, chooses a random value $v_{i}\in Z_{q}^{*}$ as $vsk_{{\mathrm{ID}}_{i}}$, and computes $vpk_{{\mathrm{ID}}_{i}}=v_{i}P$. Then A sends $vpk_{{\mathrm{ID}}_{i}}$ to *A*_I_, and updates the corresponding element $\left(vpk_{{\mathrm{ID}}_{i}},vsk_{{\mathrm{ID}}_{i}}\right)$ in the list *L*_*K*_. Otherwise, If $vpk_{{\mathrm{ID}}_{i}}\neq ⊥$, A directly sends $vsk_{{\mathrm{ID}}_{i}}$ to *A*_I_. Additionally, if *L*_*K*_ does not include $\left({\mathrm{ID}}_{i},{\mathrm{PID}}_{i},Psk_{{\mathrm{ID}}_{i}},vsk_{ID_{i}},vpk_{ID_{i}}\right)$, A makes a *CreateUser* query, sends $vsk_{{\mathrm{ID}}_{i}}$ to *A*_I_, and adds $\left({\mathrm{ID}}_{i},{\mathrm{PID}}_{i},Psk_{{\mathrm{ID}}_{i}},vsk_{ID_{i}},vpk_{ID_{i}}\right)$ to the list *L*_*K*_.
6. *ReplaceKey* query: When *A*_I_ asks for PID_*i*_, A checks whether $\left({\mathrm{PID}}_{i},Psk_{{\mathrm{ID}}_{i}},vsk_{ID_{i}},vpk_{ID_{i}}\right)$ is included in *L*_*K*_, let $vsk_{{\mathrm{ID}}_{i}}=⊥$, and replace the public key $vpk_{{\mathrm{ID}}_{i}}$ with $vpk_{{\mathrm{ID}}_{i}}^{\prime }$.
7. *H* query: When receiving an inquiry about identity ID_*i*_, A searches for triples (ID_*i*_,*r*_*i*_,*l*_*i*_) in *L*_*H*_. If (ID_*i*_,*r*_*i*_,*l*_*i*_) can be found, A returns $Q_{{\mathrm{ID}}_{i}}=l_{i}P$ to *A*_I_. Otherwise, A randomly selects $r_{i},l_{i}\in Z_{q}^{*}$, and adds (ID_*i*_,*r*_*i*_,*l*_*i*_) to *L*_*H*_, return $Q_{{\mathrm{ID}}_{i}}=l_{i}P$ to *A*_I_ finally. Specifically, if the inquiry about the challenge’s identity $I\hat{D}$ for the first time, A randomly selects $\hat{r}\in Z_{q}^{*}$, adds $\left(I\hat{D},\hat{r},bP\right)$ to *L*_*H*_.
8. *h*_1_ query: When *A*_I_ asks *h*_1_, A searches for triples $\left(r_{i},nonce,pass_{{\mathrm{ID}}_{i}}\right)$ in $L_{h_{1}}$. If $\left(r_{i},nonce,pass_{{\mathrm{ID}}_{i}}\right)$ can be found, A returns $pass_{{\mathrm{ID}}_{i}}$ to *A*_I_. Otherwise, A randomly selects $pass_{{\mathrm{ID}}_{i}}\in Z_{q}^{*}$, adds $\left(r_{i},nonce,pass_{{\mathrm{ID}}_{i}}\right)$ to $L_{h_{1}}$, and returns $pass_{{\mathrm{ID}}_{i}}$ to *A*_I_ finally.
9. *h*_2_ query: When *A*_I_ asks *h*_2_, A searches for $\left(pass_{{\mathrm{ID}}_{i}},Q_{{\mathrm{ID}}_{i}},T_{i},OTP\right)$ in $L_{h_{2}}$. If $\left(pass_{{\mathrm{ID}}_{i}},Q_{{\mathrm{ID}}_{i}},T_{i},OTP\right)$ can be found, A returns *OTP* to *A*_I_. Otherwise, A randomly selects $OTP\in Z_{q}^{*}$, adds $\left(pass_{{\mathrm{ID}}_{i}},Q_{{\mathrm{ID}}_{i}},T_{i},OTP\right)$ to $L_{h_{2}}$, and returns *OTP* to *A*_I_ finally.
10. *h*_3_ query: When *A*_I_ asks *h*_3_, A searches for $\left(\beta ,{\mathrm{ID}}_{i},T_{i}^{\circ },C_{i}\right)$ in $L_{h_{3}}$. If $\left(\beta ,{\mathrm{ID}}_{i},T_{i}^{\circ },C_{i}\right)$ can be found, A returns *C*_*i*_ to *A*_I_. Otherwise, A randomly selects $C_{i}\in Z_{q}^{*}$, adds $\left(\beta ,{\mathrm{ID}}_{i},T_{i}^{\circ },C_{i}\right)$ to $L_{h_{3}}$, and returns *C*_*i*_ to *A*_I_ finally.
11. *h*_4_ query: When *A*_I_ asks *h*_4_, A searches for (PID_*i*_,*D*_*i*_) in $L_{h_{4}}$. If (PID_*i*_,*D*_*i*_) can be found, A returns *D*_*i*_ to *A*_I_. Otherwise, A randomly selects $\mu_{i}\in Z_{q}^{*}$, let *D*_*i*_ = *μ*_*i*_, adds *D*_*i*_ to $L_{h_{4}}$, and returns *D*_*i*_ to *A*_I_ finally.
12. *h*_5_ query: When *A*_I_ asks *h*_5_, A searches for $\left(Q_{{\mathrm{ID}}_{i}},T_{i}^{\circ },j\right)$ in $L_{h_{5}}$. If $\left(Q_{{\mathrm{ID}}_{i}},T_{i}^{\circ },j\right)$ can be found, A returns *j* to *A*_I_. Otherwise, A randomly selects $j\in Z_{q}^{*}$, adds $\left(Q_{{\mathrm{ID}}_{i}},T_{i}^{\circ },j\right)$ to $L_{h_{5}}$, and returns *j* to *A*_I_ finally.
13. *h*_6_ query: When *A*_I_ asks *h*_6_, A searches for $\left(M_{i},{\mathrm{PID}}_{i},vpk_{{\mathrm{ID}}_{i}},U_{i},t_{i},h_{i}\right)$ in $L_{h_{6}}$. If $\left(M_{i},{\mathrm{PID}}_{i},vpk_{{\mathrm{ID}}_{i}},U_{i},t_{i},h_{i}\right)$ can be found, A submits *h*_*i*_ to *A*_I_. Otherwise, A randomly selects $h_{i}\in Z_{q}^{*}$, adds $\left(M_{i},{\mathrm{PID}}_{i},vpk_{{\mathrm{ID}}_{i}},U_{i},t_{i},h_{i}\right)$ to $L_{h_{6}}$, and returns *h*_*i*_ to *A*_I_ finally.
14. Sign query: When *A*_I_ asks for a signature from (PID_*i*_,*M*_*i*_), A checks eight lists: $L_{H},L_{h_{1}},L_{h_{2}},L_{h_{3}},L_{h_{4}},L_{h_{5}},L_{h_{6}},L_{K}$. If $\left({\mathrm{ID}}_{i},{\mathrm{PID}}_{i},Psk_{{\mathrm{ID}}_{i}},vsk_{ID_{i}},vpk_{ID_{i}}\right)$ is not in the list *L*_*K*_, A lets $vpk_{{\mathrm{ID}}_{i}}=⊥$, randomly selects $v_{i}\in Z_{q}^{*}$ as $vsk_{{\mathrm{ID}}_{i}}$, and computes $vpk_{{\mathrm{ID}}_{i}}=v_{i}P$. Therefore A generates $\left(vpk_{{\mathrm{ID}}_{i}},vsk_{{\mathrm{ID}}_{i}}\right)$, then adds $\left({\mathrm{ID}}_{i},{\mathrm{PID}}_{i},Psk_{{\mathrm{ID}}_{i}},vsk_{ID_{i}},vpk_{ID_{i}}\right)$ to *L*_*K*_. Otherwise, if $\left({\mathrm{PID}}_{i},Psk_{{\mathrm{ID}}_{i}},vsk_{ID_{i}},vpk_{ID_{i}}\right)$ is in *L*_*K*_, A checks $vsk_{{\mathrm{ID}}_{i}}=⊥$ and $vpk_{{\mathrm{ID}}_{i}}\neq ⊥$, this indicates that *A*_I_ has replaced the public key of the user. If $ID_{i}=I\hat{D}$, outputs failure and stops. Otherwise, A extracts the corresponding PID_*i*_ and $vpk_{ID_{i}}$ from table *L*_*K*_, finds the corresponding record for ID_*i*_ in table *L*_*K*_ and extract *l*_*i*_, finds the corresponding record for ID_*i*_ in table $L_{h_{4}}$ and extract *D*_*i*_, finds the corresponding record for ID_*i*_ in table $L_{h_{3}}$ and extract *C*_*i*_. Next, A can simulate the vehicle PID_*i*_, and generates the signature of *M*_*i*_ through the following two steps:

① chooses a random number $u_{i}\in Z_{q}^{*}$, and compute *U*_*i*_ = *u*_*i*_*P*∈*G*_1_.
② computes $h_{i}=h_{6}(M_{i},{\mathrm{PID}}_{i},vpk_{{\mathrm{ID}}_{i}},U_{i},t_{i})$, *t*_*i*_ represents the current time.

Let $S_{i}=l_{i}C_{i}D_{i}P_{pub}+y_{i}(vpk_{ID_{i}}+h_{i}U_{i})$, A outputs the signature *σ*_*i*_ = (*U*_*i*_,*S*_*i*_) for message *M*_*i*_, and returns it to *A*_I_.

It is easy to verify that the simulated signature *σ*_*i*_ = (*U*_*i*_,*S*_*i*_) mentioned above can pass the verification algorithm. Note the randomness of parameters *l*_*i*_, *C*_*i*_, *D*_*i*_ mentioned above, so the simulated signature *σ*_*i*_ = (*U*_*i*_,*S*_*i*_) is indistinguishable from real signature, that is *A*_I_ cannot distinguish whether it is a simulated signature or not.

(16) Forgery: Assuming that after answering the above inquiry, *A*_I_ can forge a valid signature $\sigma_{i}^{\prime }=(U_{i}^{\prime },S_{i}^{\prime })$ on $M_{i}^{\prime }$, and the corresponding vehicle’s identity happens to be the challenge identity $I\hat{D}$.

The following analysis shows that A can successfully solve the CDH problem by using valid signature $\sigma_{i}^{\prime }=(U_{i}^{\prime },S_{i}^{\prime })$ corresponding to the challenge identity $I\hat{D}$ forged by attackers *A*_I_. The specific methods are as follows:

Notes $\hat{e}(S_{i}^{\prime },P)=\hat{e}(D_{i}\cdot {\mathrm{PID}}_{i},P_{pub})\cdot \hat{e}(vpk_{{\mathrm{ID}}_{i}}+h_{i}U_{i}^{\prime },P_{rsu_{i}})$, A finds the corresponding *C*_*i*_, *D*_*i*_, *h*_*i*_ and $vpk_{{\mathrm{ID}}_{i}}$ in lists $L_{h_{3}},L_{h_{4}},L_{h_{6}},L_{K}$ based on identity $I\hat{D}$. Because $PI\hat{D}=bP$, *P*_*pub*_ = *aP*, we can see that $S_{i}^{\prime }=D_{i}C_{i}abP+y_{i}(vpk_{{\mathrm{ID}}_{i}}+h_{i}U_{i}^{\prime })$, then $abP=D_{i}^{-1}C_{i}^{-1}(S_{i}^{\prime }-y_{i}(vpk_{{\mathrm{ID}}_{i}}+h_{i}U_{i}^{\prime }))$ can be obtained and the CDH problem is solved.

Next, we will analyze the probability of A successfully solving the CDH problem. A winning the game requires completing the following four things:

*E*_1_ means that A will not fail during the game. *E*_2_ indicates that *A*_I_ forged a valid signature. *E*_3_ denotes the event where *A*_I_ has successfully forged a legitimate signature, resulting in A not terminating the game. *E*_4_ indicates that *A*_I_ has successfully forged a valid signature and A will refrain from terminating the game, and the forged signature corresponds to a challenged identity $I\hat{D}$.

Next, we analyze the probability of each event occurring,

$$P(E_{1})\geq {\left(1-\frac{1}{\left(q_{Psk_{{\mathrm{ID}}_{i}}}+1\right)}\right)}^{q_{Psk_{{\mathrm{ID}}_{i}}}}{\left(1-\frac{1}{\left(q_{sign}+1\right)}\right)}^{q_{sign}},P(E_{2}|E_{1})=\varepsilon ,P(E_{3}|E_{1}\Lambda E_{2})=\varepsilon P(E_{1}),$$

$$P(E_{4}|E_{1}\Lambda E_{2}\Lambda E_{3})=\varepsilon P(E_{1})\frac{1}{q_{H}}\geq \varepsilon \frac{1}{q_{H}}{\left(1-\frac{1}{\left(q_{Psk_{{\mathrm{ID}}_{i}}}+1\right)}\right)}^{q_{Psk_{{\mathrm{ID}}_{i}}}}{\left(1-\frac{1}{\left(q_{sign}+1\right)}\right)}^{q_{sign}},$$

Due to the large enough $q_{Psk_{{\mathrm{ID}}_{i}}}$, ${\left(1-\frac{1}{\left(q_{Psk_{{\mathrm{ID}}_{i}}}+1\right)}\right)}^{q_{Psk_{{\mathrm{ID}}_{i}}}+1}\to \frac{1}{e}$, Therefore, in time $t^{\prime }=t_{Succ_{A_{I}}}+(q_{c}+q_{H}+q_{h_{i}}+q_{Psk_{{\mathrm{ID}}_{i}}}+q_{vsk_{{\mathrm{ID}}_{i}}}+q_{sign})t_{M}$, A solves the CDH problem with probability $\frac{\varepsilon }{q_{H}e^{2}}$.

**Theorem 2** Under the attack of adaptive choose identity and message in a random oracle model. If an attacker *A*_II_ is capable of creating a legitimate aggregate signature within a specified time frame $t_{Succ_{A_{II}}}$ with a significant, non-negligible probability *ε* after performing *q*_*H*_ times of *H*queries, $q_{h_{i}}$ times of *h*_*i*_ (*i* = 1,2,⋯,6) queries, *q*_*c*_ times of *q*_*c*_ queries, $q_{Psk_{{\mathrm{ID}}_{i}}}$ times of *RevealPartiaPrivatelKey* queries, $q_{vsk_{{\mathrm{ID}}_{i}}}$ times of *RevealPrivateKey* queries, *q*_*sign*_ times of *Sign* queries with A. So A can completely solve the CDH problem with an undeniable probability $\frac{\varepsilon }{q_{H}e^{2}}$ in time $t^{\prime }=t_{Succ_{A_{II}}}+(q_{c}+q_{H}+\sum_{i=1}^{6}q_{h_{i}}+q_{Psk_{{\mathrm{ID}}_{i}}}+q_{vsk_{{\mathrm{ID}}_{i}}}+q_{sign})t_{M}$, where *t*_*M*_ denotes the duration required to compute scalar multiplication in the group *G*_1_ once, while *e* stands for a natural constant with a value of approximately 2.718281828459045.

The proof of Theorem 2 adopts a similar methodology as that of Theorem 1. Hence, the detailed proof process of Theorem 2 is omitted. By exploiting the well-known intractability of the CDH problem, the signature scheme proposed in this paper guarantees the infeasibility of forgery.

From the above, it can be found that the new algorithm satisfies the security requirements of VANETs, including identity privacy protection, pseudonyms, message authentication, mutual authentication, non-repudiation, untraceability, unlinkability, resistance to replay attacks, resistance to MITM (Man-in-the-Middle) attacks, resistance to impersonation attacks, resistance to simulation attacks, and user location privacy. The analysis process is analogous to that in reference [28]. Owing to space limitations, a detailed description is omitted herein.

## 7 Analysis of computing and communication costs

**Fig 1:**
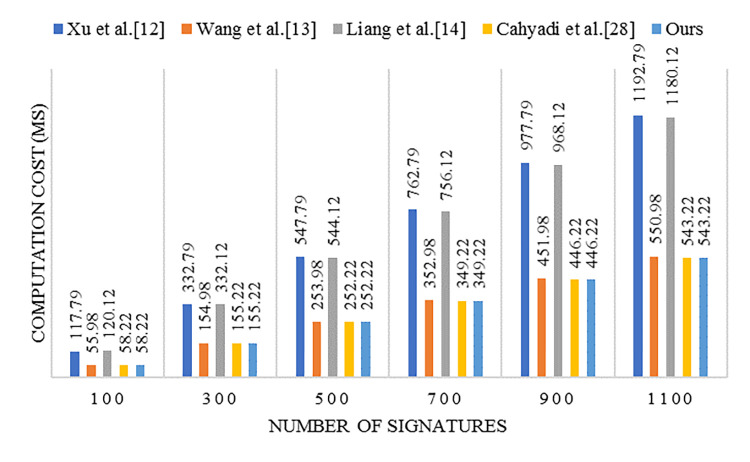
The computational cost of aggregate verification for different *n*.

This article uses third-party data to analyze the computational efficiency of some schemes. Altaf et al. [10] conducted experiments on a simulation machine using MIRACL database data to obtain the basic cryptographic operation time (Linux Mint operating system with Core-i7@3.40 GHz processor and 16 GB RAM driver). Under CDHP, the computational overhead of one-way hash function operations is very low. Therefore, the main operations considered in this article are: Bilinear-Pairing(*T*_*bp*_=3.24 *ms*), Map-to-Point in *G*_1_(*T*_*H*_=0.58 *ms*), Scalar Multiplication in *G*_1_(*T*_*m*_=0.24 *ms*), Point Addition in *G*_1_(*T*_*a*_=0.005 *ms*). Using the above data, the analysis and comparison of the computational cost results between this article and several similar schemes [12–14, 28] are presented in Table 1, where *n* represents the number of users with aggregated signatures. The security of these schemes is also shown in Table 1. The computational cost considering different numbers of messages is illustrated in Fig 1.

**Table 1:** Comparison of the computational cost and the security.

Scheme	Sign	Verify	Aggregate-Verify	Security
Xu et al. [12]	$\begin{array}{l} 1T_{H}+3T_{m}+2T_{a}\\ =1.31ms \end{array}$	$\begin{array}{l} 2T_{H}+2T_{m}+1T_{a}\\ +3T_{bp}=11.365ms \end{array}$	$\begin{array}{l} (n+1)T_{H}+2nT_{m}\\ +(3n-2)T_{a}+3T_{bp} \end{array}$	Yes
Wang et al. [13]	$3T_{m}+1T_{a}=0.725ms$	$\begin{array}{l} 2T_{m}+3T_{a}+2T_{bp}\\ =6.975ms \end{array}$	$2nT_{m}+3nT_{a}+2T_{bp}$	No
Liang et al. [14]	$\begin{array}{l} 3T_{H}+4T_{m}+2T_{a}\\ =2.71ms \end{array}$	$\begin{array}{l} 3T_{H}+2T_{m}+4T_{bp}\\ =15.18ms \end{array}$	$\begin{array}{l} (n+2)T_{H}+2nT_{m}\\ +4T_{bp} \end{array}$	Yes
Cahyadi et al. [28]	$4T_{m}+2T_{a}=0.97ms$	$\begin{array}{l} 2T_{m}+1T_{a}+3T_{bp}\\ =10.205ms \end{array}$	$2nT_{m}+nT_{a}+3T_{bp}$	No
ours	$3T_{m}+2T_{a}=0.73ms$	$\begin{array}{l} 2T_{m}+1T_{a}+3T_{bp}\\ =10.205ms \end{array}$	$2nT_{m}+nT_{a}+3T_{bp}$	Yes

From Table 1, it can be observed that our signature scheme demands only marginally more computational overhead compared to Wang et al.’s scheme [13] during the single signature generation stage, and Wang et al.’s scheme is insecure and will be vulnerable to KGC attacks. As shown in Table 1 and Fig 1, our scheme presents a computational cost comparable to that of Cahyadi et al.’s scheme [28], which surpasses other schemes in the signature verification stage. In the aggregation verification stage, it is notable that our scheme shows a slightly higher computational cost than Wang et al.’s scheme [13] when the total message count is 300 or less. However, as the message volume rises, the computational efficiency of our scheme becomes more prominent, displaying an advantage over the existing methods.

In terms of communication overhead, this article uses the relevant parameters used in reference [28]. The length of the cyclic addition group *G*_1_ is 128 bytes. The length of the multiplication group *G*_2_ is 40 bytes. The length of the timestamp is 4 bytes. The output length of a universal one-way hash function is the same as the length of the number in $Z_{q}^{*}$, both being 20 bytes. The length of traffic related messages in VANETS is 67 bytes. The communication overhead results of this article and several similar schemes [12–14, 28] are analyzed and compared is shown in Table 2.

**Table 2:** Comparison of the communication overhead for *n* messages.

Scheme	Sign	Communication overhead(byte)
Xu et al. [12]	$\left(m_{i},ID_{i},PK_{i},P_{pub},\sigma =(W_{i},V_{i})\right)$	596*n*
Wang et al. [13]	$\begin{array}{l} (m_{i},TS_{i},PK_{i}=(X_{i},R_{i}),PID_{i,j}=\{PID_{i,1,j},T_{i,j}\},\\ \sigma_{i}=(U_{i},V_{i},W_{i})) \end{array}$	859*n*
Liang et al. [14]	$\begin{array}{l} (M_{i},tt_{i},PSUID_{i}^{\prime }=\{R_{i},PID_{i},TP_{i}\},ID_{b},pk_{i},\\ \sigma_{i}=(D_{i},E_{i})) \end{array}$	735*n*
Cahyadi et al. [28]	$\left(M_{i},t_{i},PID_{i},vpk_{ID_{i}},\sigma_{i}=(U_{i},S_{i})\right)$	583*n*
ours	$\left(M_{i},PID_{i},vpk_{ID_{i}},t_{i},\sigma_{i}=(U_{i},S_{i})\right)$	583*n*

**Fig 2:**
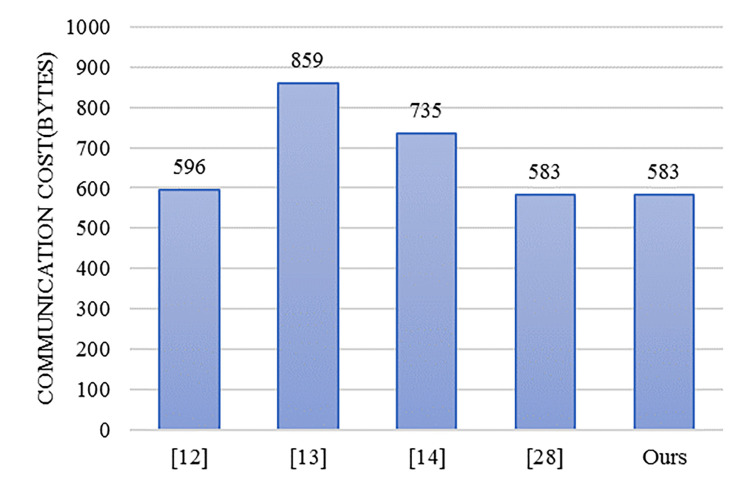
The communication overhead for a single message.

By inspecting Fig 2 and Table 2, it is revealed that our scheme has the same communication overhead as Cahyadi et al.’s scheme and surpasses the other three schemes in communication efficiency.

**Fig 3:**
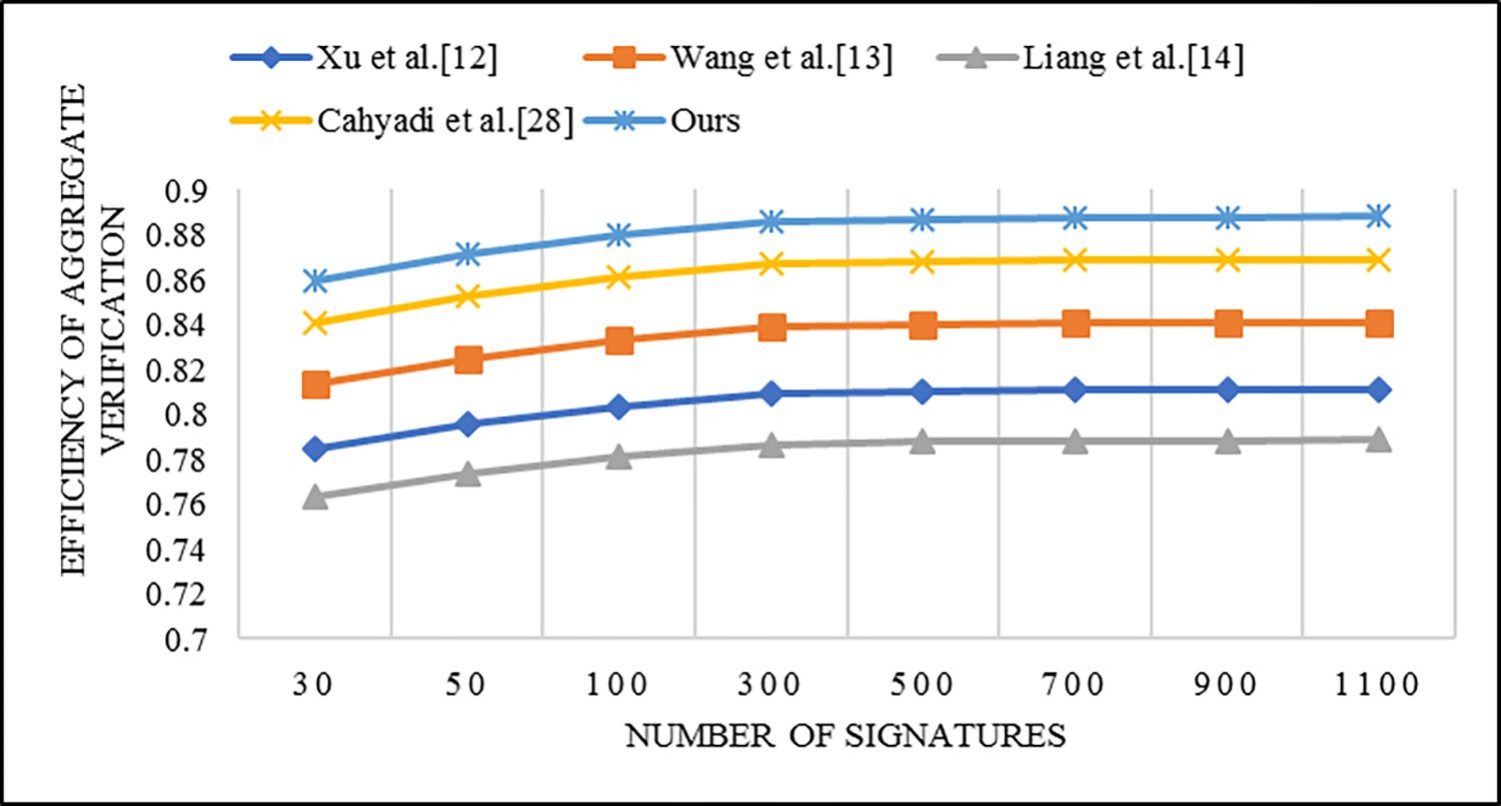
Comparison of calculational efficiency β of different schemes.

Furthermore, we take into account the calculational efficiency aspect of certificateless aggregate signature schemes. According to the definition given by Zhao et al. [43], let *K* denote the total computational cost of signature and verification for *n* messages, and *L* denote the computational cost of signature verification after aggregation. Then, the calculational efficiency is defined as β=(*K*-*L*)/*K*. The calculational efficiency β of each scheme is presented in Table 3, and the comparison results are illustrated in Fig 3. From Fig 3, it can be observed that our scheme exhibits the highest calculational efficiency.

**Table 3:** Comparison of the calculational efficiency β of different schemes for *n* messages.

Scheme	Calculational efficiency β
Xu et al. [12]	$\frac{(n-1)T_{H}+(2-2n)T_{a}+(3n-3)T_{bp}}{(3T_{H}+5T_{m}+3T_{a}+3T_{bp})n}$
Wang et al. [13]	$\frac{(2n-2)T_{bp}}{(5T_{m}+4T_{a}+2T_{bp})n}$
Liang et al. [14]	$\frac{(2n-2)T_{H}+(4n-4)T_{bp}}{(6T_{H}+6T_{m}+2T_{a}+4T_{bp})n}$
Cahyadi et al. [28]	$\frac{(3n-3)T_{bp}}{(6T_{m}+3T_{a}+3T_{bp})n}$
ours	$\frac{(3n-3)T_{bp}}{(5T_{m}+3T_{a}+3T_{bp})n}$

The aforementioned observations and analyses highlight the subtle yet crucial advantages of our algorithm regarding computational and communication overheads as well as calculational efficiency. With respect to security, our in-depth analysis has detected vulnerabilities in Cahyadi et al.’s scheme, which is prone to both public key replacement attacks and KGC attacks. Likewise, Wang et al.’s scheme is also susceptible to KGC attacks. Consequently, the improvement scheme proposed in our article outperforms the other four schemes [12–14, 28] used for comparison.

## 8 Conclusion

This article conducts a rigorous security analysis of the certificateless aggregate signature scheme for VANETs proposed by Cahyadi et al. [28]. The study reveals that Cahyadi et al.’s scheme cannot withstand two crucial security vulnerabilities: malicious KGC attack and public key replacement attack. The specific reasons for the existence of these attack methods are presented in this paper. Subsequently, this article presents an enhanced scheme aimed at strengthening against these attacks, accompanied by proof of its enhanced security. Through a comparative analysis of the computational cost, communication overhead, calculational efficiency and security of the proposed scheme with several similar schemes, the results demonstrate that our scheme exhibits superior performance in both computational and communication efficiency, along with enhanced security. The security analysis and attack methodologies described here are effective not only for the signature schemes mentioned in [28] but also possess reference value for the analysis and design of other similar signature schemes. This is especially the case for those based on bilinear pairings, as well as certificateless and certificate-based signature schemes. In future work, we will further refine and design certificateless aggregate signature schemes. This will involve considering more potential attacks, integrating reputation management mechanisms and privacy-based signature schemes, and evaluating their performance in diverse simulated and real vehicle networks.
